# The Effect of Hyperventilation Syndrome on Ionized and Serum Calcium: A Case Presentation in the Emergency Department

**DOI:** 10.7759/cureus.42310

**Published:** 2023-07-22

**Authors:** Casey McGillicuddy, Caroline Molins

**Affiliations:** 1 Emergency Medicine, AdventHealth East Orlando, Orlando, USA

**Keywords:** methamphetamines, hyperventilation syndrome, poc screening, ionized calcium, hypocalcemia

## Abstract

Hyperventilation syndrome is described as a constellation of symptoms that are typically benign but can become a medical emergency in the setting of severe metabolic derangement secondary to shifts in a patient’s pH. A 36-year-old male with a history of intravenous (IV) drug abuse presented to the emergency department (ED) in distress, complaining of diffuse muscle cramping with tetany and peripheral vasospasm. This case report discusses his initial ED testing and treatment when ionized calcium was low and serum calcium was high.

## Introduction

Hyperventilation syndrome (HVS) is described as breathing at a rate higher than what is needed to maintain normal levels of the blood’s partial pressure of oxygen in arterial blood (PaO_2_) and partial pressure of carbon dioxide in arterial blood (PaCO_2_). This increase in respiratory rate can be induced by the body’s stress response [1]. Mild to moderate symptoms of HVS can be vague and described as perioral numbness, tingling sensations in the distal extremities, dyspnea, near syncope, and palpitations. More severe symptoms such as tetany, arrhythmias, and hyperreflexia result from respiratory alkalosis that can cause extracellular ion shifts [2].

In the emergency department (ED), hyperventilation and its commonly seen clinical symptoms are often overlooked because they can be seen as a diagnosis of exclusion [3]. In HVS, an acute rise in pH can result in ionized calcium binding to albumin and an intracellular shift of potassium, resulting in hypokalemia [4-5]. These electrolyte imbalances can contribute to fatal electrocardiographic (EKG) changes that must be addressed in the emergency department [4]. This report will highlight the case of a 36-year-old male with a history of intravenous (IV) drug abuse who presented to the emergency department (ED) in distress, complaining of diffuse muscle cramping with tetany and peripheral vasospasm. This case report discusses the clinical presentations of hyperventilation syndrome, initial ED testing, and treatment in the setting of ionized calcium and serum calcium abnormalities.

## Case presentation

A 36-year-old male with a history of intravenous drug abuse presented to the ED via ambulance for diffuse muscle cramping approximately 30 minutes after using methamphetamines. The patient used the words "locked up" to describe how he felt in his muscles and mentioned that he felt like he could not move. It was noted on the physical exam that the patient was anxious, tachypneic, with lungs that were clear to auscultation bilaterally, and tachycardic with no murmurs. In addition, he had delayed capillary refill (>2 seconds) on his hands and feet and displayed physical symptoms of tetany on his bilateral upper extremities during blood pressure measurements. Laboratory and point-of-care (POC) metabolic panels and blood gases were ordered. Arterial blood gas (ABG) reported a pH of 7.667 and a paCO_2_ of 18.9 mmHg, while initial venous blood gas (VBG) reported a pH of 7.643 and a pCO_2_ of 22.8 mmHg. These blood gases point to an acute respiratory alkalosis. His labs showed low ionized calcium (1.0 mmol/L) on the POC metabolic panel, high serum calcium (11.2 mg/dL), normal potassium (4 mmol/L), elevated creatinine (2.49 meq/L), and an elevated creatine kinase (CK) at 273 units/L. The patient was given 1 mg of lorazepam and 10 mg of calcium gluconate intravenously (10 ml ampule of 10% calcium gluconate) to treat anxiety and hypocalcemia, respectively. Within 30 minutes of medication administration, the patient’s respiratory rate returned to normal, and he reported reduced anxiety. After two hours of observation in the emergency department, laboratories were repeated and reported that the patient’s pH, pCO_2_, and ionized calcium had normalized. His repeat serum bicarbonate was elevated, revealing a chronic, compensated metabolic acidosis. Tables 1-2 show the initial and repeat laboratory findings.

**Table 1:** The patient’s initial and repeat blood gas values ABG: arterial blood gas; VBG: venous blood gas; pCO_2_: partial pressure of carbon dioxide; p0_2_: partial pressure of oxygen; HCO_3_: bicarbonate; mmHg: millimeters of mercury; meq: milliequivalents; L: liter; NR: no range

	Initial ABG	Normal values	Initial VBG	Normal value	Repeat VBG (+ 2 hour)
pH	7.667	7.35-7.45	7.643	7.32 - 7.4	7.356
pCO_2_ (mmHg)	18.9	35-45	22.8	NR	54
pO_2_ (mmHg)	166.4	80-105	25	NR	27.4
HCO_3_ (meq/L)	21.6	22-26	24.7	22-29	30.3

**Table 2:** The patient’s initial and repeat basic metabolic panels POC: point-of-care; meq: milliequivalents; L: liter; mmol: millimole; g: grams; * denotes from POC only

	Initial level	Normal values	Repeat POC (+2 hours)	Normal values
Sodium (mmol/L)	136	135-145	135	138-144
Potassium (mmol/L)	4	3.5-5	3.6	3.5-4.5
Chloride (mmol/L)	90	98-110	96	98-104
CO_2_ (meq/L)	22	24-32	30	23-30
Serum calcium (mg/dL)	11.2	8.5-10.5	-	
Ionized calcium (mmol/L)	1.00*	1.15-1.33	1.28*	1.15-1.33
Creatinine (meq/L)	2.49	0.6-1.20	2.1	0.51-1.19
Albumin (g/dL)	5.3	3.2-5.5	-	

In addition, after two hours of observation in the ED, the patient reported the resolution of all his symptoms, including muscle cramps, dyspnea, and chest pain. The patient’s capillary refill normalized on physical exam, and there was no longer tetany with blood pressure measurements.

## Discussion

This case illustrates a patient with tachypnea, tachycardia, diffuse muscle cramping with tetany on blood pressure measurement, and peripheral vasospasm whose likely diagnosis is hyperventilation syndrome (HVS). Panic attacks, anxiety, asthma, exercise, severe pain, or acute blood loss can cause HVS [3]. Symptoms that characterize HVS are tachypnea, dyspnea, carpopedal spasm, tachypnea, tachycardia, anxiety, and numbness of the extremities, among others, in mild to moderate cases. It can progress with symptoms of tetany, arrhythmias, and hyperreflexia resulting from respiratory alkalosis that can cause extracellular ion shifts [1,2]. In many cases, HVS is a diagnosis of exclusion; therefore, they may have tests conducted, such as laboratory testing, basic metabolic panels, and blood gases [3]. In HVS, an acute rise in pH can result in ionized calcium binding to albumin and an intracellular shift of potassium resulting in hypokalemia, as was seen in the case presented [4-5]. In some cases of HVS, the patient’s respiratory alkalosis will be complicated by metabolic acidosis, which may help guide the patient’s management. The mainstay of treatment for HVS syndrome is the treatment of the underlying condition. In this case, HVS was likely methamphetamine-induced, which the patient admitted to taking before presenting to the ED. Short-term side effects of methamphetamines, like those of other stimulants, include agitation, anxiety, decreased appetite, tachypnea, tachycardia, elevated blood pressure, and body temperature [6]. In this case, anxiolytics such as benzodiazepines were used to reduce the patient’s anxiety and respiratory rate, likely caused by the drug use.

In this case, POC labs ultimately changed the management of this patient. In the emergency department, POC labs are important as they are relied on for quick turnaround times and correlating lab-generated values [7]. This, however, is not the case with calcium. Calcium in the extracellular fluid exits in three distinct ways: protein-bound (40%), ionized (50%), and anion-bound (10%) [8]. A basic metabolic panel measures serum calcium, which comprises bound calcium, and a POC calcium panel only measures the ionized form of calcium, which may not correlate to the serum level. Serum calcium has been shown to be a poor indicator of clinical symptoms since only ionized calcium is freely exchanged across cells. Thus, an ionized calcium level is critical in patients at risk of hypocalcemia, as this is not included in a typical basic metabolic panel, which is usually not included in typical basic metabolic panels. It is also important to recognize that in alkalotic states, ionized calcium’s affinity for albumin increases [2,5]. Rapidly bound calcium decreases the amount of free calcium, which decreases the threshold at which the cardiac membrane needs to depolarize and puts the patient at risk for arrhythmias. In this patient, the hyperventilation likely caused alkalosis and decreased ionized calcium, which manifested clinically with carpopedal spasm and tetany [2]. Treating the low-ionized calcium with calcium gluconate, thereby addressing the electrolyte imbalances caused by the patient’s alkalotic state, improved his symptoms.

## Conclusions

Hyperventilation syndrome (HVS) is an important diagnosis that may require treatment of metabolic and underlying conditions in severe cases. This phenomenon requires careful consideration as it can cause electrocardiographic changes and arrhythmias. Additionally, it is critical to understand the role of POC labs in contrast to serum lab studies, especially in regard to their difference in accuracy when treating critically ill patients.
